# Prevalence of endosalpingiosis and other benign gynecologic lesions

**DOI:** 10.1371/journal.pone.0232487

**Published:** 2020-05-13

**Authors:** Jan Sunde, Morgan Wasickanin, Tiffany A. Katz, Emily L. Wickersham, D. O. Emilie Steed, Novae Simper

**Affiliations:** 1 Department of Obstetrics and Gynecology, Madigan Army Medical Center, Tacoma, WA, United States of America; 2 Division of Gynecologic Oncology, Baylor College of Medicine, Houston, TX, United States of America; 3 Department of Pathology Madigan Army Medical Center, Tacoma, WA, United States of America; 4 Department of Medicine, Texas Tech University Health Sciences Center, Lubbock, TX, United States of America; CHU Clermont-Ferrand, FRANCE

## Abstract

Endosalpingiosis, traditionally regarded as an incidental pathological finding, was recently reported to have an association with gynecologic malignancies. To determine the prevalence of endosalpingiosis, we evaluated all benign appearing adnexal lesions using the Sectioning and Extensively Examining-Fimbria (SEE-Fim) protocol, and queried the pathology database for the presence of endosalpingiosis, gynecologic malignancy, endometriosis, Walthard nests, and paratubal cysts. Using the SEE-Fim protocol, the prevalence of endosalpingiosis, endometriosis, Walthard nests, and paratubal cysts were 22%, 45%, 33%, and 42% respectively, substantially higher than previously reported. All lesions were observed to increase with age except endometriosis which increased until menopause then decreased dramatically. Among specimens including ovarian tissue, the prevalence of implantation of at least one lesion type was ubiquitous in patients age 51 and older (93%). The clinical significance of endosalpingiosis should be a continued area of research with larger trials assessing prevalence, factors affecting incidence, and association with malignancy. Our findings contribute to elucidating the origin of ectopic lesions and gynecologic disease risk.

## Introduction

Endosalpingiosis (ES) is the ectopic peritoneal presence of glands lined by tubal-type epithelium. It differs from endometriosis (EM) in that it has ciliated glandular epithelium, no endometrial stromal component, and does not exhibit an inflammatory response. ES is an understudied area largely because it was traditionally viewed as an incidental finding, resulting in a limited understanding of its etiology and clinical significance. Recently, in a retrospective chart review, ES has been reported to be associated with gynecological malignancy. The 2016 study identified 838 cases of ES out of approximately 60,000 gynecologic specimens (a prevalence of 1–2%), of which 354 (42% of ES cases) had a concurrent gynecologic malignancy[1]. This association with cancer suggests that ES may be a more concerning finding than previously reported, especially since it is now widely accepted that serous ovarian carcinomas do not originate from ovarian surface epithelium, but from serous epithelium arising either in the Fallopian tube (FT), or an ectopic location. This paradigm shift occurred over the last 2 decades, and now has significant supporting evidence[1–11].

The presence of other benign cellular implants in the peritoneal cavity (EM, Walthard nests (WN), and paratubal cyst (PTC)) have also been associated with malignancy. EM is reported to have a 3-fold statistically significant increase in the risk of ovarian carcinomas, particularly endometrioid and clear cell carcinomas[12]. Transitional cell neoplasms (Brenner tumors) were significantly associated with WN in 50% of cases, while WN only appeared in 28% of controls[13]. Another report found WN present in 43% of patients with Brenner tumors, and also reported an association with mucinous tumors[14]. Transitional cell carcinoma, an entity that rarely arises outside the bladder has been reported within the FT as primary paratubal transitional cell carcinoma, and has also been associated with WN and/or PTC[15]. We sought to explore ES prevalence using a more comprehensive protocol and associations with malignancy, as well as other ectopic tissues present in the pelvis, including EM, WN, and PTC.

## Materials and methods

This study was approved by the Institutional Review Board at Madigan Army Medical Center (MAMC) which determined this work to be exempt. This was a retrospective study designed to determine the prevalence of ES prior to and after implementing the use of the Sectioning and Extensively Examining of the Fimbriated end (SEE-Fim) protocol for all gynecologic specimens in our institution’s Department of Pathology.

In July 2016, the pathologists at MAMC began consistently assessing for, and reporting the presence of benign appearing small adnexal lesions on all gynecologic specimens. This practice change was adopted to more accurately capture a prevalence for ES, after the above described association between ES and malignancy raised concerns that ES could be a malignant precursor. The SEE-Fim protocol is traditionally utilized only for the examination of specimens obtained from patients at risk for tubal/ovarian cancer [16,17].

### Histology

Tissue specimens were macroscopically assessed after fixation in 10% buffered formalin, then serial sectioned. The entire tube and ovary were submitted for histologic analysis. The distal 2 cm of the tube were amputated and sectioned sagitally into 4 sections. The remainder of the tubes were sectioned at 2- to 3-mm intervals. The ovaries were sectioned at 2- to 3-mm intervals. Sectioned specimens were stained with hematoxylin and eosin as per standard pathology practice. ES was defined as nests of tubal type ciliated epithelium located in places which they would not be expected to be found (i.e. along the serosal surface of the FT, or on other gynecologic organs or pelvic structures). The distinction between PTC and larger foci of ES was based on gross appearance and size. Serous cystadenomas were diagnosed as larger, more complex structures lined by primarily cuboidal epithelium. Ovarian inclusion cysts (OIC) were only included if they were lined by Müllerian type epithelium. Mesothelial invaginations of surface projections were not included in this study. Endometriosis was diagnosed only if a combination of endometrial glands and stroma were present. WN were reported when nests of cells resembling urothelium were identified.

Query of the pathology database was performed to identify all patients who underwent gynecologic surgery (removal of FT, ovaries, uteri, and/or peritoneum) between Jan 2015—Dec 2015 and July 2016—June 2017 at MAMC. Specimen type, patient age, surgical indication, presence and site of ES (ovarian surface involvement and OIC were reported separately), and presence and type of malignancy, and other tissue findings were collected by the investigating pathologist. Data was then de-identified prior to release to study investigators.

Collected data was broken into two time periods, Jan 2015 through Dec 2015 and July 2016 through June 2017, to compare prevalence with and without using the SEE-Fim protocol. Sub-cohort analyses were performed by age, and by the types of tissue present in the specimen. Data was also examined for associations of ES with gynecologic malignancy and other benign lesions. The prevalence of other benign lesions; EM, WN and PTC was also assessed.

### Statistical analysis

Subgroup analyses were done by age, dividing groups by decade. Since several 10 year subgroups had inadequate numbers of lesions for statistical analysis, the women were divided into 3 larger subgroups, ≤30, 31–50, and >50. Chi-square test was used to compare the distribution of the absolute frequencies of ES in the subgroups compared to the entire group. All statistical analyses were performed with https://www.socscistatistics.com/tests/chisquare/.

## Results

**Fig 1:**
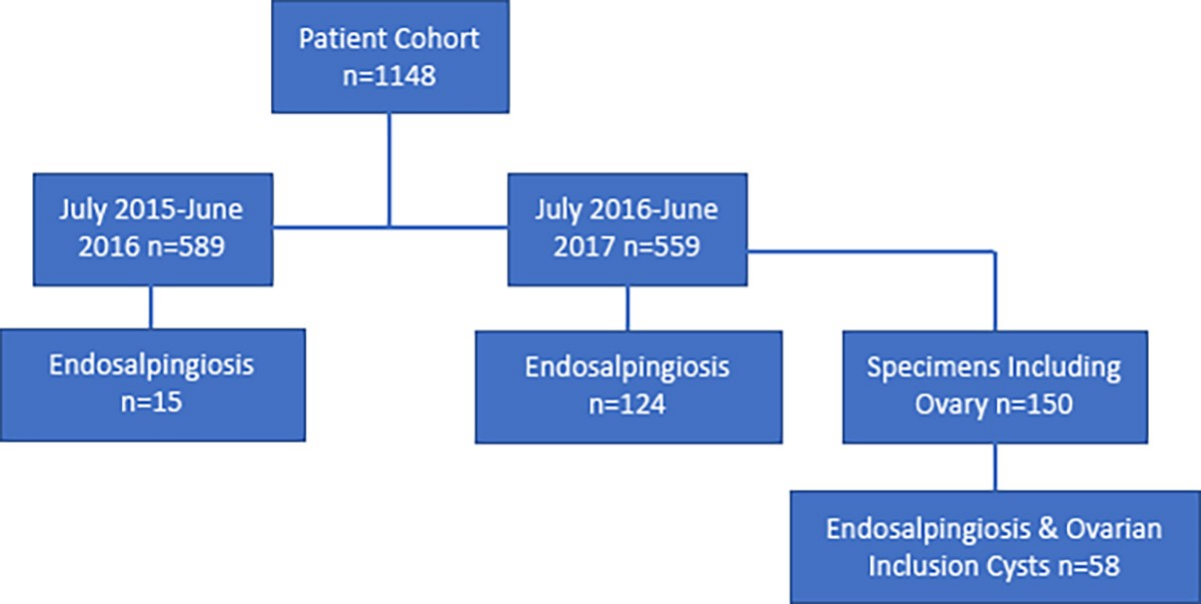
Flow diagram of tissue selection process.

One thousand one hundred and forty-eight patients with gynecologic specimens were identified, of which 589 were collected between July 2015 and June 2016, and 559 were collected between July 2016 and June 2017. During the period of July 2015-June 2016, prior to the implementation of SEE-Fim, 15 cases of ES were reported, and during July 2016-June 2017, using the SEE-Fim protocol, 124 cases were reported (14 of which were OIC), a prevalence of 2.54% and 22.15%, respectively (p < 0.00001, Table 1, Fig 1). The SEE-Fim protocol is currently the most thorough evaluation method of gynecologic pathological specimens that include adnexae in use.

**Table 1:** Endosalpingiosis prevalence (Chi square statistic).

	Percent	P value
*Time Period*		
2015–2016 (prior to SEE-Fim) N = 589	2.54	
2016–2017 (utilizing SEE-Fim) N = 559	22.15	< 0.001
*ES Prevalence Total*		
±30 years of age N = 152	6.6	
> 30 years of age N = 407	28.0	< 0.001
*ES Prevalence in FT*		
30 years of age N = 118	5.08	
> 30 years of age N = 372	22.04	< 0.001
*Women >50 years of age with ovarian tissue present N = 41*		
** ES**	**65.85**	
Cancer	46.34	
ES + Cancer	73.68	
PTC	68.29	
WN	58.54	
No reported lesions	7.32	
*Women 31–50 years of age with ovarian tissue present N = 73 (Chi square compares 31–50 vs >50)*		
** ES**	**36.99**	**0.003**
EM	30.14	
EM + ES	31.82	
EM without ES	29.41	
PTC	42.47	
PTC without ES	20.55	

For the remainder of the analysis, only specimens from the arm of the study using SEE-Fim were evaluated. In the group of patients whose samples were evaluated using SEE-Fim, their age ranged from 8–82 years, and the indications for the procedure performed were recorded (Table 2). The most common indication was abnormal uterine bleeding 31.8% (178/559). A significant proportion of patients, 27.4%, underwent sterilization by salpingectomy as they were premenopausal with no indication for oophorectomy, providing only FT specimens, while a minority of patients also had ovaries removed (22%). Some patients underwent procedures for multiple indications.

**Table 2:** Demographics and indications for surgery in all patients.

Surgical Indications	Percentage of Indications (%)
	N = 559
Abnormal Uterine Bleeding	31.8(including menorrhagia & HMB& postmenopausal)
Sterilization	27.4
Pelvic Pain	9.1
Fibroid	7.3
Adnexal Mass	6.3
Ectopic	6.3
Ovarian Cysts	5.4
Endometriosis	3.8
Prolapse	3.2
Dysmenorrhea	2.7
Infertility	2.3
Uterine Cancer	2.0 (uterine cancer& endometrial cancer & endometroid adc)
Hyperplasia	1.1 (EMBx, simple, and complex)
Breast Cancer	0.7
Incontinence	0.5
Lynch Syndrome	0.4

A subset of patients were assigned more than one indication.

All patients were between 8–82 years of age and in the subgroup utilizing SEE-Fim (n = 559)

ES prevalence was 4.7 fold higher (p < 0.00001) in women over 30 years old (28%) compared to women 30 years old and younger (6.6%, Table 1). Subdividing age groups further did not yield enough patients to evaluate for statistical significance and is presented in S1 Table.

Patient age at the time of surgery could potentially have affected which tissues were obtained, so we did several subset analyses looking at cohorts that included ovaries, and separately, FT as part of the pathological specimen. We analyzed the presence of ES based on the location where it was reported, aiding in minimizing sampling bias and thereby a better assessment of the true prevalence of ES. A large number of patients, particularly at younger ages, had only FT submitted, so these were evaluated separately to better assess change in prevalence with age. Malignancy was the indication for a large proportion of specimens in post-menopausal patients, which has previously been reported to be associated with ES. This may have led to sampling bias in older patients as well.

One-hundred twenty- three specimens (22% of all cases) included ovarian tissue and in these specimens, the prevalence of ES was 46%. The prevalence of ES in the subgroup of specimens including ovaries was higher in older women, similar to the larger cohort. The prevalence in the oldest population (>50, 66%) was approximately double that of the younger two groups which displayed similar prevalence (33% and 37%), and was significantly higher (p < 0.003) in women over 50 (66%) compared to women 31–50 (37%). We did not evaluate the prevalence difference in the cohort of youngest women due to the small sample size (n = 9, Table 3).

**Table 3:** Prevalence of benign lesions stratified by age. (Chi square statistic).

	Total, n (%)	30	31–50	> 50	P value	P value
					<30 vs 31–50	31–50 vs >50
ES (with ovaries)	57/123 (46.3)	3/9 (33.3)	27/73 (37.0)	27/41 (65.9)	NS	0.003
ES in FT	88/490 (18.0)	6/118 (5.1)	66/314 (21.0)	16/58 (27.6)	0.001^†^	NS
EM (with ovaries)^a^	30/150 (20.0)	6/36 (16.7)	22/73 (30.1)	2/41 (4.9)	NS	0.002
PTC	208/495 (42.0)	37/123 (30.1)	139/314 (44.3)	32/58 (55.2)	0.003^†^	NS
WN	162/490 (33.1)	31/118 (26.3)	100/314 (31.9)	31/58 (53.5)	NS	0.002

^a^Additional EM cases are represented as endometriomas were also included

^†^chi-square statistic with Yates correction

To further evaluate whether the prevalence of ES changes with age and to minimize sampling bias based on the tissue type evaluated, a subgroup analysis based on the presence of ES of the FT as part of the pathology specimen was performed. This eliminated a frequent site of implantation, the ovary, but allowed for comparison of age cohorts that had different rates of oophorectomy. Again a significantly higher prevalence was observed in older women (p < 0.00008, Table 1). The prevalence of ES of the FT appeared to continue to increase by decade after age 30 until menopause when we looked at ten year intervals, with the prevalence in the 31–40 cohort of 19% (37/200) and the 41–50 cohort, at 25% (29/114), respectively. This was an interesting finding, although the cohorts were not large enough to evaluate for statistical significance. We also report the prevalence of ES ± OIC by decade in S1 Table which documents a similar trend.

A subgroup analysis was also conducted in women who underwent sterilization with only FT specimens obtained, which amount to 28% of all patients undergoing gynecologic surgery for sterilization purposes. Of the 152 specimens collected from women 30 and younger, 50% (76/152) were removed for sterilization, whereas in the 31–50 group, only 20% (81/407) of procedures were for sterilization. In the subgroup of women who underwent sterilization with only FT removed, ES was identified in 5% (4/76) of women under 30, and 9% (7/81) of women age 31–50. The numbers were too small to determine if this increase with age was significant. The prevalence of ES in women age 31–50 undergoing sterilization, 9% (7/81), was statistically significantly lower than ES in the larger cohort in women age 31–50 who had their procedure for indications other than sterilization, 25% (60/237).

We also evaluated the location of the reported ES lesions, to better understand its distribution (Table 4). We limited this evaluation to women who had ovary specimens available because the ovaries were the only site of involvement in a number of patients. Women under age thirty rarely had ovaries removed, but ES was present in 3/9 (33%). We examined lesion location separately in women age 31–50 and over 50. Twenty-one percent (73/343) of women age 31–50 had their ovaries removed, while 64% (41/64) of women over 50 had ovaries removed, which was a statistically significant difference (P = 0.00001). Thirty-seven percent (27/73) of women between 31–50 had ES, with a higher prevalence in the menopausal women, 66% (27/41). ES involving the ovaries was substantially higher in both women 31–50, 59% (16/27), and women over 50, 89% (24/27) (Table 4).

**Table 4:** Location of endosalpingial lesions.

Percentage	31–50	> 50 (64)	P value(chi-square)
	N = 343	N = 64	31–50 vs >50
Specimens with Ovaries (%)	21.3	64.1	0.001
ES in Specimens with Ovaries	31–50 n = 73	>50 n = 41	
ES Prevalence (%)	37.0	65.9	0.003
ES, OIC (%)	48.1	81.4	0.023^†^
ES, surface only with no OIC (%)	22.2	7.4	NS
ES, FT only (%)	40.7	7.4	0.011^†^
ES, multiple sites (%)	11.1	51.9	0.003^†^
ES, other sites (peritoneum, serosa) (%)	7.4	11.1	NS

^†^chi-square statistic with Yates correction

We considered whether lesions found on the ovary involved the surface only, or had developed into OIC. Forty-eight percent (13/27) of women age 31–50 had ES involvement of the ovarian surface only, whereas only 7% (2/27) of the women >50 had only ovarian surface involvement. Forty-one percent (11/27) of women age 31–50 had ES involvement of the FT only, compared to 11% (3/27) of the older women. In women over 50 ES was higher in frequency and multiplicity. Women age 31–50 had multiple site involvement in 11% (3/27) of cases, while menopausal women had multiple sites in 52% (14/27) of cases (P = 0.001268). There were similar percentages of specimens having involvement of other sites such as the uterine serosa or peritoneum in both age groups, 7% (2/27) vs 11% (3/27) (Table 4).

The association of ES and other benign lesions with cancer was examined in women over 50 who had specimens including ovaries (n = 41). Of 41 post-menopausal specimens including ovaries, 66% had ES and 46% had gynecologic malignancy (Table 1). More than half of patients with ES also had OIC. Seventy-four percent of specimens with a gynecologic malignancy had concurrent ES(including OIC), higher than in prior reports, and a similar number of women in this subgroup had PTC (68%) and WN (59%). The number of patients in each subgroup were too small to assess for statistical significance. Of note, only 7% of specimens had no reported benign lesions, i.e., 93% of women harbored at least one benign lesion of any type.

We evaluated patients who had FT specimens available for the prevalence of PTC, since PTC are associated with the tubes and may be pedunculated ES lesions. Several specimens from patients under 30 had PTC excised separately from their FT, so we included these specimens in this assessment. The denominator in this cohort was therefore slightly larger than the FT only cohort used above. We found that the prevalence of PTC increased significantly with age <31 vs 31–50 vs >50 (30% vs 44% vs 55%), similar to ES (Table 3, p = 0.000078).

We considered the effect on ES prevalence if PTC is considered a type of ES, also arising from the FT. In women in the 31–50 age group with ovary specimens available, the prevalence of ES was 27/73 (37%), PTC were associated with ES in 16/27 cases, and were found alone in 15/73 (21%) cases, therefore the prevalence of ES including PTC increases to 42/73 (58%) (Table 1).

There were significantly more WN lesions in specimens from the women 30 and under (26%) compared to the number of ES lesions (7%, Table 1). The prevalence of WN was higher than other benign lesions in young women, and did not appear to increase significantly with age until menopause (p = 0.001554) (Table 3).

We also looked at the presence of serous cystadenomas and cystadenofibromas in our entire cohort, since they have an epithelium similar to the FT epithelium. There were 19 such lesions, 5 of which were removed by cystectomy, and were not expected to have associated lesions. Of the remaining 14, all were associated with ES and PTC.

EM is another non-malignant finding reported in gynecologic specimens, and is associated with pain symptoms in many patients, leading to interventions. We evaluated all women with any ovarian tissue present (n = 150), including ovarian cystic lesions, to evaluate for EM prevalence, since cystic endometriotic lesions (endometriomas) can be removed without ovarian epithelium to preserve ovarian tissue. This may have decreased the prevalence of EM in combination with ES, since the whole ovary was not assessed for ES in all patients. We found 26% (28/109) of all patients under 50 years old had EM, and a higher percent of patients age 31–50 (30%) then those under 30 (17%). This finding was not statistically significant, given the small numbers of patients. We also noted that the prevalence of EM drops dramatically after menopause (5%), which is expected since it is a hormone responsive condition (Table 3). In the subgroup age 31–50, we found EM concurrent with ES in 32% of women which is comparable to the previously reported 37%[1].

## Discussion

This retrospective review has demonstrated a markedly higher prevalence of ES than is reported in the literature. Over a 12 month period, the prevalence of ES among gynecologic specimens including ovarian tissue was 37% in women over 30, and even higher at 66% after menopause. This is in the same range as the 49% prevalence of ES found in normal appearing pre-menopausal ovaries in a recent pathology review [18] although one must question whether the indication for surgery in that review may have affected prevalence, as 25 of the 57 patients evaluated in their study were women having risk-reducing salpingo-oophorectomy. Prior studies have not described the location and relative frequency of ES in the peritoneal cavity. When the location of ES was considered, over half of the specimens had lesions only on the ovaries, so the true prevalence of ES *in vivo* may be closer to the prevalence we found in pre-menopausal women >30 with ovarian tissue present (37%), as opposed to the prevalence reported for all patients (22%). Another finding of our study was that ES was most commonly found on the ovary as OIC. Three of the 9 (33%) ovaries removed in women ≤30 had ES, so it is possible that the true prevalence in young women ≤30 is higher than what we found in all young women (7%), and the ES prevalence in younger women at the time of laparoscopy previously reported to be 7.6%[19]. The pathology report of normal ovaries cited previously did not report prevalence based on age [18]. The prevalence in both young and old patients may be affected by sampling bias, with hesitation to perform ovarian biopsies in patients interested in fertility, and a substantial proportion of post-menopausal patients with a cancer diagnosis, leading to more thorough pathological evaluation. Our institution’s prevalence prior to implementing SEE-Fim for all gynecologic specimens was similar to that which was previously reported in the literature (2.5%), so the higher prevalence found when using SEE-Fim is likely due to the enhanced evaluation of the adnexae. We believe that the 37% we found when using SEE-Fim in patients with ovaries removed may approximate the true prevalence of ES, and that it could be higher, but additional studies with larger numbers will be needed to confirm these findings, and to conduct subgroup analyses to further understand the epidemiology and pattern of distribution of ES. The most accurate way to assess the true prevalence of ES would be surgical evaluation of a random cohort of women with biopsies to confirm the diagnosis, but such a study is not feasible.

Recent studies have reported a prevalence of ES at 1.5%[1] - 3.5%[20], and a significant association with gynecologic cancers, particularly after menopause[1], raising the question whether ES could be a malignant precursor lesion. Another nationwide registry cohort study subsequently also reported an association of ES with ovarian cancer [21]. We also found ES (9/19), as well as OIC (10/19) present in menopausal patients with cancer approximately 50% of the time. By using the SEE-Fim protocol for thorough examination of all specimens including ovaries, we found the ES prevalence was much higher than the previously reported 1.5%, at 37% and 66% in women age 31–50 and after menopause, respectively. Given the much higher prevalence of ES that we found in post-menopausal patients with approximately three quarters of the ES patients having cancer, and our sample size, we were unable to make a significant association between ES and cancer. Sampling bias may have increased the prevalence of patients with both ES and cancer, since most gynecologic surgery after menopause is due to cancer. It is unclear whether prior reports of an association between ES and cancer may be a result of sampling bias due to thorough evaluation of cancer specimens, with no control group of ES patients without cancer. It will be difficult to determine whether there is a true association of ES and cancer, or whether both conditions just happen to be found frequently in surgical specimens after menopause, without knowing what the baseline prevalence of ES is in post-menopausal women. Another review of a hospital pathology database in 2012 which included 110 patients found that 44/110 (40%) of ES occurred in post-menopausal women, without a significant association with cancer [22]. We found a smaller percentage (25%) of ES occurred in post-menopausal patients, presumably because more ES was reported due to improved sampling in pathology specimens from younger women. A finding noted previously described is that ES is found in multiple locations significantly more often in women after menopause.

The increase in prevalence of ES with age parallels that of EM up to menopause, at which time EM prevalence falls dramatically, and ES prevalence appears to increase further. While EM is known to be responsive to hormones and expected to decrease with menopause, little is know about ES, and it appears to have no response to menopausal hormone changes with persistence after menopause noted.

ES has also been reported to have a prevalence of 33% in association with serous borderline tumors (SBT) in 2 large cohort studies, and a prevalence as high as 70% noted in women with recurrent SBT in a third SBT study[23–25]. One of the studies looked at a large cohort of over 1000 patients and found a significantly higher ES prevalence in patients with recurrent SBT, 33%, vs 9% in the patients without recurrence[24]. The study evaluated patients from 1978 to 2002, which was prior to the era of SEE-Fim, when FTs were thought to be a possible site of origin for SBTs. The sampling of the FT may have been less extensive, contributing to the low prevalence reported in the group without recurrence. The report describes that 60% of patients with recurrent SBT had stage II/III disease at initial diagnosis, so the question of sampling error as a source of the significant difference arises, in light of the prevalence of over 30% in the general population reported in our work, since that rate is comparable to the ES prevalence in their recurrence cohort. We suspect that ES has been under-reported in the past, because the traditional pathological evaluation of normal appearing FT was not as robust as the SEE-Fim protocol.

Another recent report has found ES with genetic mutations that are identical to those present in the SBT [26]. ES in patients with SBT had KRAS or BRAF mutations in 52% (10/21 patients) of cases, whereas ES not associated with ovarian tumor rarely had mutations (1/13 patients). Given the higher ES prevalence in recurrent SBT, and these genetic mutations, there may be a role for evaluation of benign appearing ES lesions to identify a group of patients at risk for developing SBT or low grade serous carcinoma.

Increasing ES with age was noted in our study, which is consistent with what has been noted in mouse models of OIC, that have found an increase in number with age and number of ovulations.

This is true in a normal mouse strain,[27] as well as four transgenic mouse models, which have demonstrated an increase in the number and size of OIC [27–30], suggesting that the specific genes perturbed in these models may play a role in the development of ES. A recent paper sought to use lineage tracing to demonstrate that OIC is derived from oviductal origin using a genetically engineered mouse model[31].

Interestingly, our data show that the occurrence of ES could possibly be related to the presence of gynecologic conditions that lead to surgery. Sterilization is typically performed on women who do not have another indication for gynecologic surgery, so we compared women who underwent surgery for sterilization with women undergoing surgery for other reasons. The prevalence of ES of the FT in women age 31–50 having their procedure for sterilization (9%, 7/81) was statistically significantly lower than FT involvement in women of the same age undergoing surgery for other indications (21%, 66/314, p = 0.01). Understanding why women with ES have a higher rate of gynecologic pathological diseases may help develop better prevention strategies.

We also evaluated the prevalence of other benign lesions. PTC are reported to be mostly simple serous cysts, and these benign lesions have been reported in up to 25% of women with adnexal masses[32,33]. We report a substantially higher prevalence of PTC at 42%, including in the cohort of women under 30 (30%), which was double or more than the prevalence of ES in each age group. While the origin of PTC is unclear, it also increased with age, and there appears to be some hormonal effect. Reports of surgeries of young female patients found to have PTC describe patients near menarche or after, but not young girls, unless they had precocious puberty[34–36].

Endometriosis prevalence (20%) was at the high end of the previously reported range of 2–20% [37,38], and it also was noted to increase from 16% in women 30 and under, to 30% in women 31–50, similar to previous findings [38], with a dramatic drop to only 5% after menopause. The true EM prevalence may be higher, since typically, symptomatic EM patients present for care, gynecologists are hesitant to biopsy ovaries in infertile patients, and some of the cases found in our study were patients treated for other conditions. Endometriosis has been previously associated with EOC, specifically endometrioid and clear cell histology [39]. Prior studies evaluating EM prevalence were not conducted using the SEE-Fim protocol, therefore our data may be a closer approximation to true prevalence, and older studies reporting an association between EM and cancer may need to be re-evaluated using a more accurate prevalence rate. The prevalence of EM was dramatically lower following menopause, demonstrating the importance of estrogen on this disease process, in contrast to the other benign lesions evaluated.

Walthard nests, a non-Müllerian tissue, are also more prevalent in our patients than previously reported (20%) [13], with lesions in 26% of young women, increasing to 53% in the menopausal cohort. This increase with age has not previously been reported. While uncertain, one theory concerning the origin of WN is metaplasia at the FT-peritoneal junction [40]. Their reported prevalence of transitional cell metaplasia at the junction of the columnar epithelium and serosa was 20%, with an additional 35% having lesions of the fimbriae and surrounding peritoneum in a cohort of patients with a mean age of 52. These numbers are similar to the WN prevalence we found in menopausal women, of 53%.

The origin of benign ectopic tissue in the female pelvis remains uncertain, with 3 competing theories, metaplasia of local epithelium, sloughing and ectopic growth of epithelia from their site of origin, and vestigial cells deposited along the migratory track in embryologic development, what is called the “Secondary Müllerian System”[11,41]. Metaplasia of the ovarian surface epithelium has fallen out of favor as the origin of Müllerian carcinomas, although metaplasia of the FT is still considered a possible source of WNs [42]. This is an area of active research, as shown by a recent paper published looking at genetically modified mice [31], that demonstrated that at least some portion of OIC comes from the oviductal epithelium, since they defined ES/OIC as cystic structures with cells that expressed genes that are only active in the oviductal epithelium, and these ES lesions were present in 14% of adult mice. Interestingly, they concluded that OIC does not come from the oviduct, and that OIC does not increase with age in mice, but their experiments were clearly limited by inadequate numbers, with 1/9 mice having ES at 1 month post treatment, 2/20 mice having ES at 6 months, and 4/20 mice at 12 months. Another variant of these theories is called the “dualistic model” which ascribes the source of ovarian cancer to both a metaplastic origin, and a Fallopian tube origin. [43,44]. This concept is based on differential gene expression in different serous ovarian cancers, and comparison with gene expression in ovarian surface epithelium (OSE) vs normal FT epithelium. The cell of origin for the gene expression of “normal” tissue is critical for these papers [45], and the frequent presence of ES we found on the ovarian surface raises the question whether there may have been alterations of the OSE gene expression data by ES present on the ovarian surface of “normal” ovaries. A similar question is a concern in the experimental modeling of Zhang, et al, i.e., whether there may have been oviductal cells present when they developed their OSE model by use of Cre induction 3 days prior to salpingectomy [44]. More research is clearly needed to further elucidate the initial steps in the process of ovarian cancer development.

There are some limitations of our prevalence study. It was a retrospective review, therefore patient information was limited to that provided to pathology. We were unable to assess the influence of factors such as parity, hormone use or prior tubal ligation on the presence of ectopic epithelium. Further research would be expected to show that these factors which affect ovarian cancer risk should decrease the presence of all ectopic epithelial types, and may be helpful in determining the origin of ectopic Müllerian tissues. We believe the prevalence reported here is close to the true prevalence of ES, although the true number may be even higher. Several factors could increase prevalence: the type and extent of tissues removed at surgery may affect the true prevalence; the percentage of patients evaluated using thorough pathological evaluation (a small subset of specimens were not submitted in their entirety in our study); the pathology evaluation technique—more lesions may have been identified with even more tissue sampling.

We have demonstrated that ES, and other benign ectopic tissues, are much more common than previously reported, with ES prevalence in as high as 37% in women age 31–50. It appears from our data that ES is present in approximately 2/3 of menopausal patients, both with and without cancer. We did not have adequate numbers to show whether there is an association of ES and gynecologic cancer or not, since the control group should be patients with ES and no gynecologic cancer. We believe the mere presence of ES should not be considered a cancer precursor, since the prevalence of ES is so common, and the presence of ES with cancer may be coincidental. Our study does not elucidate the mechanism of ectopic presence of these tissues in the female pelvis, and further studies, both epidemiological and experimental are needed.

## Supporting information

S1 Table Prevalence of endosalpingiosis by age in decades. (DOCX)

S1 Data (XLSX)
