# Correction: Prevalence of endosalpingiosis and other benign gynecologic lesions

**DOI:** 10.1371/journal.pone.0248443

**Published:** 2021-03-04

**Authors:** The PLOS ONE Staff

The fifth author’s name is incorrect. The correct name is: Emilie Steed. The publisher apologizes for the error. The correct citation is: 1. Sunde J, Wasickanin M, Katz TA, Wickersham EL, Steed E, Simper N (2020) Prevalence of endosalpingiosis and other benign gynecologic lesions. PLoS ONE 15(5): e0232487. https://doi.org/10.1371/journal.pone.0232487
