# Supplementary material for: Prevalence of endosalpingiosis and other benign gynecologic lesions
Source: PLoS One. 2020 May 13;15(5):e0232487. doi: 10.1371/journal.pone.0232487 (PMC7219775; doi:10.1371/journal.pone.0232487)
Supplement: S1 Table — (DOCX) [file pone.0232487.s001.docx]

**Supplemental Table 1**. Prevalence of endosalpingiosis by age in decades

| **Age** | **ES (%)** | **ES + OIC (%)** | **Total n** |
| --- | --- | --- | --- |
| *ES in specimens with ovaries* |  |  |  |
| 8-30 | 2 (5.56) | 4 (11.11) | 36 |
| 31-40 | 9 (23.68) | 12 (31.58) | 38 |
| 41-50 | 14 (40.00) | 15 (42.86) | 35 |
| 51-82 | 19 (46.34) | 27 (65.85) | 41 |
| *ES in FT* |  |  |  |
| 8-30 | 6 (5.08) |  | 118 |
| 31-40 | 37 (18.50) |  | 200 |
| 41-50 | 29 (25.44) |  | 114 |
| 51-82 | 16 (27.59) |  | 58 |
| *ES in all specimens* |  |  |  |
| 8-30 | 7 (4.61) | 10 (6.58) | 152 |
| 31-40 | 45 (20.45) | 48 (21.82) | 220 |
| 41-50 | 35 (28.46) | 37 (30.08) | 123 |
| 51-82 | 23 (35.94) | 30 (46.88) | 64 |
